# Pinhole-free TiO_2_/Ag_(O)_/ZnO configuration for flexible perovskite solar cells with ultralow optoelectrical loss†

**DOI:** 10.1039/c9ra00042a

**Published:** 2019-03-19

**Authors:** Eunwook Jeong, Soohyun Bae, Jong Bae Park, Seung Min Yu, Donghwan Kim, Hae-Seok Lee, Jongjoo Rha, Young-Rae Cho, Jungheum Yun

**Affiliations:** Surface Technology Division, Korea Institute of Materials Science Changwon Gyeongnam 51508 Republic of Korea jungheum@kims.re.kr +82-55-280-3570 +82-55-280-3515; Department of Materials Science and Engineering, Pusan National University Busan 46241 Republic of Korea yescho@pusan.ac.kr +82-51-514-4457 +82-51-510-2389; Department of Materials Science and Engineering, Korea University Seoul 02841 Republic of Korea ramun16@korea.ac.kr +82-2-3290-3713; Jeonju Center, Korea Basic Science Institute Jeonju Jeonbuk 54907 Republic of Korea; KU-KIST Green School, Graduate School of Energy and Environment, Korea University Seoul 02841 Republic of Korea

## Abstract

Perovskite solar cells (PSCs) fabricated on transparent polymer substrates are considered a promising candidate as flexible solar cells that can emulate the advantages of organic solar cells, which exhibit considerable freedom in their device design thanks to their light weight and mechanically flexibility while achieving high photocurrent conversion efficiency, comparable to that of their conventional counterparts fabricated on rigid glasses. However, the full realization of highly efficient, flexible PSCs is largely prevented by technical difficulties in simultaneously attaining a transparent electrode with efficient charge transport to meet the specifications of PSCs. In this study, an effective strategy for resolving this technical issue has been devised by proposing a simple but highly effective technique to fabricate an efficient, multilayer TiO_2_/Ag_(O)_/ZnO (TAOZ) configuration. This configuration displays low losses in optical transmittance and electrical conductivity owing to its completely continuous, ultrathin metallic Ag_(O)_ transparent electrode, and any notable current leakage is suppressed by its pinhole-free TiO_2_ electron transport layer. These features are a direct consequence of the rapid evolution of Ag_(O)_ and TiO_2_ into ultrathin, completely continuous, pinhole-free layers owing to the dramatically improved wetting of metallic Ag_(O)_ with a minimal dose of oxygen (*ca.* 3 at%) during sputtering. The TAOZ configuration exhibits an average transmittance of 88.5% in the spectral range of 400–800 nm and a sheet resistance of 8.4 Ω sq^−1^ while demonstrating superior mechanical flexibility to that of the conventional TiO_2_ on ITO configuration. The photocurrent conversion efficiency of flexible PSCs is significantly improved by up to 11.2% thanks to an optimum combination of optoelectrical performance and pinhole-free morphologies in the TAOZ configuration.

## Abstract 2

A TiO_2_/Ag_(O)_/ZnO configuration is developed for flexible perovskite solar cells to provide a pinhole-free electron transport layer and a transparent electrode.

## Graphical abstract

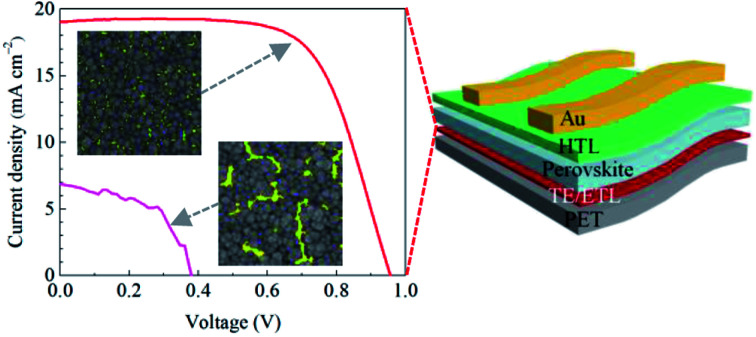

## Introduction

Mechanically flexible solar cells (FSCs) supported on polymer substrates have attracted increasing attention for their potential to be implemented in bendable, portable, and even wearable devices, and their structural durability against mechanical deformation is of primary importance.^1–3^ The technical attractiveness of FSCs has been further strengthened by their potential to be fabricated using continuous roll-to-roll processes, which offer the merits of high throughput and low cost.^4–6^ Thus far, efforts to technologically advance FSCs have concentrated mainly on increasing the photocurrent conversion efficiency (PCE) by improving light harvesting and charge-carrier collection.^7–11^ However, this task is still technically challenging due to choice constraints on the materials that absorb photons and transfer charge carriers. Attaining a high PCE requires a photoactive material that efficiently absorbs photons and rapidly transfers charge carriers to external circuits. However, the type of device implemented for FSCs is currently limited to organic solar cells (OSCs), which provide low charge-carrier extraction efficiencies owing to the inferior properties of polymer-based photoactive materials, including lower photon absorbance, faster charge-carrier recombination, and a shorter exciton diffusion length than their inorganic counterparts.^12,13^ In addition to these issues, the charge-carrier extraction efficiency of FSCs is seriously degraded by the use of transparent electrodes (TEs). The currently available amorphous-oxide-based TEs, predominantly represented by amorphous indium tin oxides (a-ITOs), exhibit poor electrical conductivities when they are integrated into FSCs supported on polymer substrates. An amorphous phase inevitably forms for most oxide TEs, including ITO TEs, deposited on heat-sensitive polymers at substrate temperatures clearly below the deformation temperature of the polymers, about 120 °C, with very limited exceptions.^14^ Efforts to improve the electrical conductance of amorphous ITOs by increasing their thickness have proven to be inadequate due to the detrimental influences of increasing thickness on the optical transparency owing to simultaneous increases in light absorbance and reflection, both of which result in a loss in the incident light available for light harvesting.^14,15^ Furthermore, increasing the ITO thickness is generally believed to escalate the chance of conductance failure in ITO TEs with the catastrophic development of cracks through film-type ITO domains when subjected to mechanical stresses if the polymer substrate is deformed.

Efforts to improve the performance of FSCs thus need to be directed toward overcoming the aforementioned issues with FSCs, which are mainly attributed to the inherent limitations of OSCs and ITO TEs. In this regard, perovskite solar cells (PSCs) offer clear advantages for use in FSCs over OSCs because PSCs exhibit a high photon absorption coefficient,^16,17^ high charge-carrier mobility,^18^ and a long charge-carrier diffusion length^19–21^ combined with feasibility for relatively low-temperature fabrication processes.^22,23^ High PCEs even compared with those of conventional bulk Si solar cells have routinely been reported for PSCs utilizing crystalline TCOs fabricated on glass substrates.^24,25^ However, such promising photocurrent conversion performance has not been attained for PSCs fabricated on highly flexible, heat-sensitive polymer substrates utilizing a-ITOs as the TE.

To circumvent the performance limitations of a-ITOs coated on heat-sensitive polymer substrates, oxide–metal–oxides (OMOs) are a promising substitute for a-ITO TEs in FSCs because they simultaneously exhibit exceptionally high electrical conductance and mechanical flexibility.^14,26^ The multilayer configuration of OMOs enables integrating charge-carrier extraction and charge transport functions into a single OMO unit by designing the top oxide layer as either an electron or hole transport layer (ETL or HTL, respectively), and the sandwiched thin metal layer serves as the main conduction path. From the electrical perspective, Ag is the best choice among metal candidates available for OMOs because its electrical resistivity (1.68 μΩ cm), the lowest among known metals, is about two orders of magnitude lower than that of a-ITO.^15^ To exploit this promising electrical property of Ag in OMOs, a continuous layer geometry must be attained for Ag with the minimum possible thickness to establish sufficient electrical paths along its longitudinal plane while decreasing the optical losses attributed to light absorption and reflection. Such optical losses can be largely suppressed by decreasing the minimum Ag thickness required to attain a continuous Ag layer, *i.e.*, by improving the morphology of the Ag thin film. This task depends on Ag wetting, which is strongly influenced by the chemical and structural status of the underlying oxide.^14^ Therefore, the choice of the underlying oxide material, in addition to the Ag deposition conditions, is a critical parameter for determining the minimum possible thickness of the continuous Ag layer. Choosing an inadequate oxide material as the bottom layer in OMOs significantly delays the formation of a continuous Ag layer and degrades the performance of OMOs.^14^ Exactly these same effects are observed when utilizing a top TiO_2_ layer as the ETL in an OMO configuration.^27,28^ TiO_2_ is well recognized for its role in transferring electrons from the photoactive layer to the electrode.^22,29^ Many reports have heavily emphasized improving the performance of the TiO_2_ ETL by suppressing the formation of micro- and nanoscopic pinhole defects, which cause catastrophic current leakages and seriously degrade the performance of PSCs.^23,29–33^ Nevertheless, attaining a pinhole-free TiO_2_ ETL in OMOs is difficult, especially when the TiO_2_ ETL is deposited on an Ag layer. However, this issue with OMOs has not been systematically investigated to make them fully applicable to highly efficient, flexible PSCs.

This study aims to design a highly efficient OMO structure that provides an ETL and TE with excellent performance, even surpassing that of conventionally available choices, for flexible PSCs fabricated on heat-sensitive poly(ethylene terephthalate) (PET) substrates. This goal was achieved by sequentially fabricating (i) a highly transparent, completely continuous Ag TE with an ultrasmall thickness of less than 10 nm and (ii) a pinhole-free TiO_2_ ETL on the Ag layer. Here, a completely continuous, ultrathin Ag TE was formed by applying a unique technique based on gas-additive-mediated metal growth, which was recently developed to improve the wettability of noble metals on chemically heterogeneous oxides in metal sputtering processes at room temperature.^34–40^ A minimal dose of an impurity gas was artificially introduced to improve Ag wetting by successfully preventing any notable degradation in the inherent characteristics of the noble metal.^34,35,39,40^ Gas-additive-mediated metal growth can improve the optical and electrical performance of Ag layers by decreasing the minimum thickness required to form a continuous layer. In addition, this growth strategy enables designing an efficient TiO_2_ ETL that suppresses serious leakage currents through nanoscopic pinholes. When a pinhole-free TiO_2_ layer was successfully coated on a completely continuous, ultrathin Ag layer, the TiO_2_ on Ag on ZnO (TiO_2_/Ag/ZnO) configuration provided sufficient ETL and TE characteristics for flexible PSCs fabricated on PET substrates. The resulting PSCs exhibited much higher PCEs than PSCs utilizing a conventional TiO_2_ on a-ITO configuration.

## Results and discussion

**Fig. 1:**
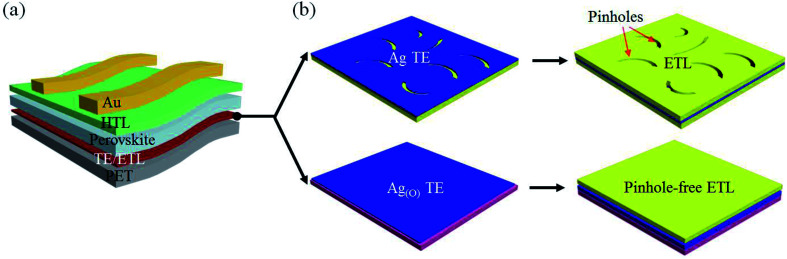
Schematic illustration of (a) the device architecture of a PSC fabricated on a flexible, heat-sensitive PET substrate by utilizing TiO_2_ and Ag as the ETL and TE, respectively, and (b) a comparison of the conventional TiO_2_/Ag/TiO_2_ (top) and proposed TiO_2_/Ag_(O)_/ZnO (bottom) structural configurations, with an emphasis on pinhole suppression in the top TiO_2_ ETL.

The material choice and structural arrangement of the OMO were optimized to facilitate highly efficient TE and ETL features that are applicable to flexible PSCs in the following configuration: Au electrode/HTL/photoactive perovskite layer/OMO/PET substrate (Fig. 1a). For the OMO structure utilizing a sandwiched Ag layer and a top TiO_2_ layer as the material candidates for the TE and ETL, respectively, special attention was paid to fabricating pinhole-free Ag and TiO_2_ layers at their minimum possible thicknesses. In this study, increasing the wettability of the Ag TE on the bottom oxide layer was proven to enhance the performance of the top TiO_2_ ETL, as schematically illustrated in Fig. 1b. When the bottom oxide layer was not carefully selected to promote Ag wetting, the significant inclusion of pinholes was routinely observed in the ultrathin Ag TEs. The pinhole-containing morphology of the Ag TE was verified to directly affect pinhole formation in the top TiO_2_ ETL subsequently deposited on the Ag TE, whereas no such pinhole-containing morphology was observed in either the Ag TE or TiO_2_ ETL by virtue of the strong Ag wetting. This wetting was achieved by growing oxygen-dosed Ag, Ag_(O)_, on a bottom oxide layer that promoted Ag wetting *via* oxygen-additive-mediated growth. The TE and ETL characteristics were expected to be significantly improved by OMOs utilizing the pinhole-free Ag and TiO_2_ layers.

**Fig. 2:**
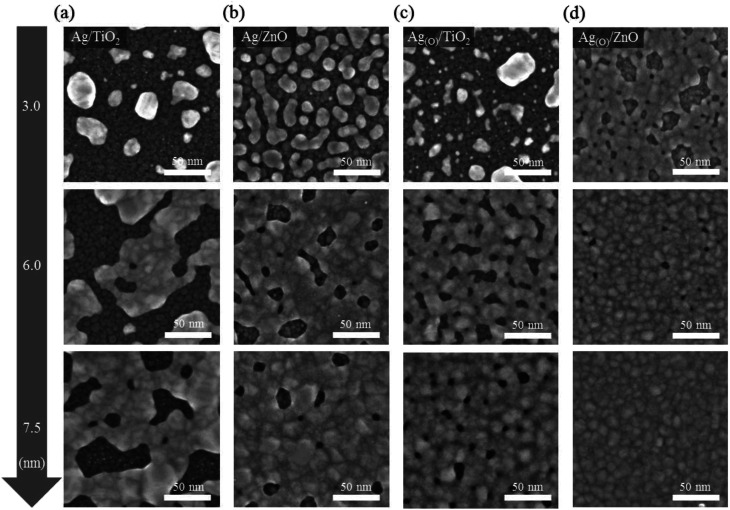
Morphological comparison of Ag and Ag_(O)_ deposited on two different bottom oxide layers: TiO_2_ and ZnO. The FE-SEM images showed the highly magnified planar surfaces of Ag and Ag_(O)_ in different configurations: (a) Ag on TiO_2_ (Ag/TiO_2_), (b) Ag on ZnO (Ag/ZnO), (c) Ag_(O)_ on TiO_2_ (Ag_(O)_/TiO_2_), and (d) Ag_(O)_ on ZnO (Ag_(O)_/ZnO). Images of the morphologies of Ag and Ag_(O)_ were captured at different nominal thicknesses: 3.0, 6.0 and 7.5 nm, from top to bottom.

Ultrahigh-resolution field-emission scanning electron microscopy (FE-SEM) images (Fig. 2) show the distinct morphologies of Ag and Ag_(O)_ deposited on TiO_2_ and ZnO as different choices for the bottom oxide layer. When Ag_(O)_ was fabricated *via* oxygen-additive-mediated growth using a reactive sputtering process at room temperature and an inlet gas mixture of Ar : O_2_ = 45 : 4 sccm, the oxygen dose in the Ag_(O)_ layer was determined to be about 3 at% using X-ray photoelectron spectroscopy (XPS). Ag_(O)_ was confirmed to be highly metallic, and no notable oxide phases were observed for this minimal oxygen level, with no negative shift in the binding energy of Ag 3d_5/2_ peak relative to that of pure Ag (Fig. S1a†). This result was also supported by the fact that the peak position and shape of the M_4_N_45_N_45_ and M_5_N_45_N_45_ Auger peaks do not change (Fig. S1b†). The oxide-phase-free nature of Ag_(O)_ was further verified by X-ray diffraction (XRD). Specifically, its crystallographic Ag(111) and the total absence of Ag_2_O and AgO peaks indicated that it was highly metallic (Fig. S2†), which is in good agreement with the results in the literature.^35,39^ Although all growth cases of Ag and Ag_(O)_ TEs surveyed in this study fulfilled the three-dimensional (3D) (Volmer–Weber) growth mode, the wettabilities of Ag and Ag_(O)_ TEs on different bottom oxide layers were clearly different. The lowest wettability was observed for pure Ag grown on a bottom TiO_2_ layer, *i.e.*, Ag on TiO_2_ (Ag/TiO_2_) (Fig. 2a). The coalescence of Ag nanoclusters (or nanoparticles) was seriously delayed, which inhibited the formation of a continuous layer of Ag on the TiO_2_ layer because the surface of the TiO_2_ was not completely wetted by Ag, even when the Ag thickness was increased to 7.5 nm. The wettability of the Ag TE on the bottom oxide layer was clearly improved by replacing TiO_2_ with ZnO (Fig. 2b). Although pinholes still existed in the 7.5 nm-thick Ag TE on a ZnO layer (Ag/ZnO), the number and size of pinholes were significantly lower than those of Ag/TiO_2_.

The improvement in the wettability of Ag on ZnO can be mainly understood as a result of the improved adhesion of Ag atoms to the underlying oxygen atoms at the Ag–ZnO interface.^14^ According to the standard heat of formation of oxides, Zn–O bonding in ZnO is much weaker than Ti–O bonding in TiO_2_.^41^ However, these weaker bonds increase the availability of the topmost oxygen atoms to Ag atoms at the Ag–ZnO interface *via* the redox (reduction–oxidation) reaction driven by charge transfer,^42^ as previously reported for Au on oxides.^43–45^ Furthermore, the clear crystallographic difference observed between TiO_2_ and ZnO layers is noteworthy. The TiO_2_ deposited at room temperature appeared to be amorphous, whereas the ZnO deposited under the same conditions exhibited high crystallinity with a (0002)-preferred orientation. Considering the good crystallographic matching between Ag(111) and ZnO(0002), using the ZnO layer to grow Ag may provide favorable circumstances for developing energetically stable Ag clusters with a (111)-preferred orientation during the first stages of Ag growth. This orientation decreases the interfacial free energy and thus improves Ag wetting on ZnO, as reported for Cu grown on ZnO.^38^

The improved wettability of Ag_(O)_ was observed even on the TiO_2_ layer thanks to the positive contribution of oxygen additives to Ag wetting on the bottom oxide layer (Fig. 2c). The working mechanism and effect of oxygen additives in the oxygen-additive-mediated growth of noble metals including Ag and Cu have been demonstrated elsewhere in detail with solid experimental and numerical evidence,^38,39^ but the results can be illustrated by a simple scenario related to controlling the free energies of Ag clusters on oxides during the very early stages of growth. When the growth of Ag clusters on TiO_2_ and ZnO layers is understood as the growth of a high-surface-energy noble metal on a low-surface-energy oxide with a weak adhesion at the Ag-oxide interface, any decrease in the surface free energy of Ag favors the generation of stable Ag clusters with significantly lower atomic diffusion and/or migration tendencies, as proposed by Bauer *et al.*^46,47^ The surface free energy of metal clusters has been reported to decrease at oxygen dose levels far below the thermodynamically favorable stoichiometric concentration of metal oxidation.^48,49^ Thus, the improved wettability of Ag_(O)_ on the TiO_2_ and ZnO layers can be explained by the decreased surface free energy of the Ag clusters with oxygen additives. The best design to maximize the wettability of Ag_(O)_ was achieved by applying a thin layer of ZnO as the bottom oxide (Fig. 2d). As a result, a completely continuous Ag_(O)_ TE with a thickness of 7.5 nm was obtained without the noticeable presence of pinholes.

**Fig. 3:**
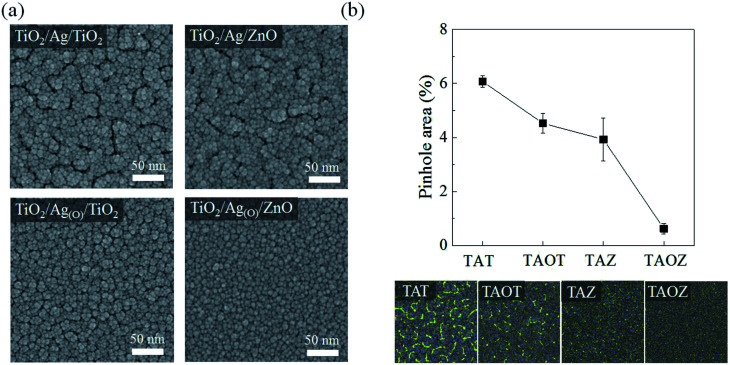
Pinholes distributed in the top TiO_2_ ETLs in different OMO structural configurations. (a) FE-SEM images showing the planar surfaces of 20 nm-thick top TiO_2_ ETLs in different OMOs: TiO_2_/Ag/TiO_2_ (TAT), TiO_2_/Ag/ZnO (TAZ), TiO_2_/Ag_(O)_/TiO_2_ (TAOT), and TiO_2_/Ag_(O)_/ZnO (TAOZ). The thicknesses of the Ag and Ag_(O)_ TEs were both fixed to 7.5 nm, whereas those of the bottom TiO_2_ and ZnO layers were fixed to 5 nm. (b) Quantitative difference between the percent surface areas of pinholes in the top TiO_2_ ETLs of different OMOs, which were determined from the FE-SEM images using image processing software. The solid line is for visual guidance.

Planar FE-SEM images demonstrated the clear morphological distinctions between TiO_2_ ETLs belonging to different OMO structural configurations: TiO_2_/Ag/TiO_2_ (TAT), TiO_2_/Ag/ZnO (TAZ), TiO_2_/Ag_(O)_/TiO_2_ (TAOT), and TiO_2_/Ag_(O)_/ZnO (TAOZ) (Fig. 3a). Highly dense, relatively large, irregular stripe-like pinholes were observed in the 20 nm-thick TiO_2_ ETLs of the TAT and TAZ configurations. These pinholes indicate that the nanoscopic gaps in the 7.5 nm Ag layers were not completely filled by the TiO_2_ ETLs subsequently deposited on the Ag layers although for the most part, the volume of these gaps was filled by the TiO_2_ (Fig. S3†). The pinholes probably passed through the TiO_2_ ETL and directly connected with the gaps in the Ag TE. Fewer pinholes formed in the TiO_2_ ETL with the TAOT configuration and were minimized in TAOZ. When the overall surface area of pinholes was determined by image processing the planar FE-SEM images of the TiO_2_ ETLs (Fig. 3b), the total pinhole area was minimized in the TAOZ configuration because the wettability of Ag_(O)_ was maximized on the ZnO layer. The nearly pinhole-free morphology of the TAOZ configuration was maintained even for the 10 nm TiO_2_ ETL (Fig. S4†), which indicated that the complete wetting of Ag_(O)_ on ZnO was a decisive factor in achieving a pinhole-free TiO_2_ ETL.

**Fig. 4:**
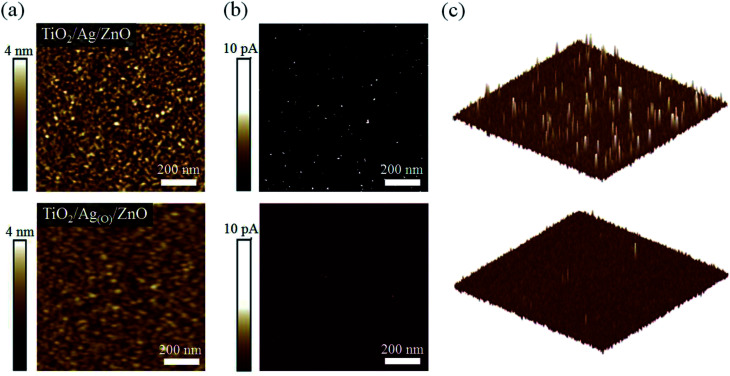
Current leakage distribution through nanoscopic pinholes in top TiO_2_ ETLs. (a) 2D morphological scan images of 20 nm TiO_2_ ETLs in the TAT and TAOZ configurations captured using tapping-mode AFM. Corresponding (b) 2D and (c) 3D current scan images of the ETLs captured using conductive-mode AFM.

Because the pinholes in the TiO_2_ ETLs might serve as sources of current leakage, thereby leading to significant current losses, successfully suppressing the formation of pinholes was expected to increase the charge separation efficiency of the TiO_2_ ETL in the TAOZ configuration relative to that of ETLs in different OMO configurations. To confirm the stripe-like pinholes as the main source of current leakage in TiO_2_ ETLs, atomic force microscopy (AFM) was performed on 20 nm TiO_2_ ETLs in two different OMO configurations: TAZ and TAOZ (Fig. 4) in the topographic and corresponding conductive modes. Holes observed in the two-dimensional (2D) topographic image (Fig. 4a) of the TiO_2_ ETL in the TAZ configuration were equated with spots of strong current flow detected in the 2D and 3D conductive AFM profiles (Fig. 4b and c). These current flow spots undoubtedly served as significant sources of current leakage by bypassing the TiO_2_ ETL. In contrast, very few spots with a number density of 3–4 μm^−2^ were observed in the ETL in the TAOZ configuration. The difference in the numbers of current flow spots between TAZ and TAOZ was in accordance with the difference in pinhole densities. Although more current flow spots were observed when the thickness of the TiO_2_ ETL in the TAOZ configuration was decreased to 10 nm, the current intensities of the spots were clearly much weaker than those of the spots observed in the ETL in TAZ (Fig. S5†).

**Fig. 5:**
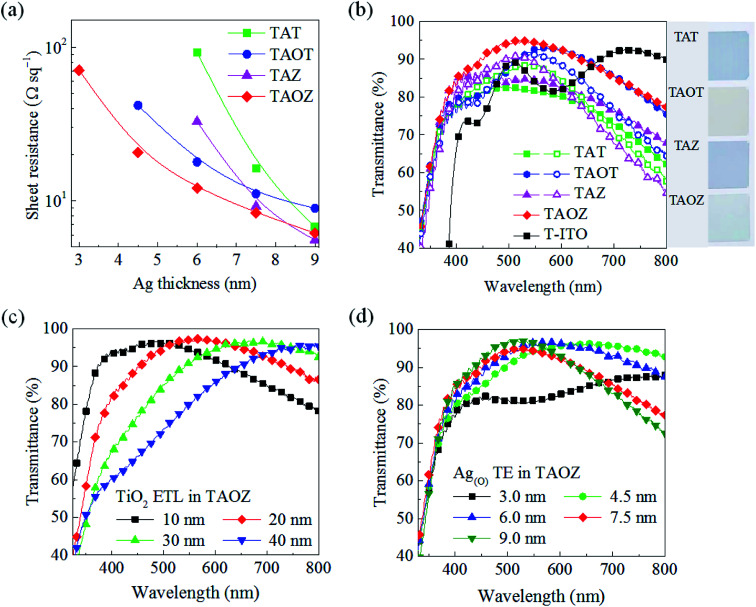
Electrical and optical characteristics of OMOs fabricated on flexible PET substrates. (a) Sheet resistance as a function of the thickness of Ag and Ag_(O)_ TEs in OMOs with 5 nm-thick bottom and 20 nm-thick top oxides. Solid lines are for visual guidance. (b) Comparison of total transmittance spectra over the spectral range of 330–800 nm of OMOs with different Ag and Ag_(O)_ thicknesses: 7.5 nm (solid symbols) and 12 nm (open symbols), and 20 nm TiO_2_/160 nm a-ITO (T-ITO). Right: visual comparison of the optical transparency of OMOs with 6 nm Ag or Ag_(O)_ TEs. (c) Total transmittance spectrum of TiO_2_/6 nm Ag_(O)_/5 nm ZnO (TAOZ) for various top TiO_2_ thicknesses. (d) Total transmittance spectrum of 20 nm TiO_2_/Ag_(O)_/5 nm ZnO (TAOZ) for various Ag_(O)_ thicknesses.

In addition to suppressing pinholes in the TiO_2_ ETL, the completely continuous layer morphology of the ultrathin 7.5 nm Ag_(O)_ TE also provided an excellent opportunity to optimize the optoelectrical characteristics of the TAOZ configuration. The fast decrease in the sheet resistance of the TAOZ configuration during the very early growth stages was clearly distinguished from its counterparts. This low sheet resistance of 8.4 Ω sq^−1^ was clear evidence of the sufficient electrical paths in the 7.5 nm Ag_(O)_ TE in the TAOZ configuration (Fig. 5a). Its total transmittance was much higher than those of other OMOs over the visible spectral range of 400–800 nm, owing to its fast morphological evolution into a continuous layer (Fig. 5b). Meanwhile, the optical transmittances of other OMOs were severely decreased as a result of photon losses. These loss were mainly due to strong optical absorbance by the incompletely coupled metal clusters because Ag and Ag_(O)_ TEs in the TAT, TAZ, and TAOT configurations did not form a continuous layer at this thickness (Fig. S6†). Here, the transmittance spectra of the OMOs were determined by subtracting the transmittance of the PET substrates. The superior optical transmittance of the TAOZ configuration relative to those of other OMOs was still effective, even when the transmittance spectra included the spectrum of the PET substrate (Fig. S7†).

Further increasing the thickness of Ag and Ag_(O)_ TEs above the minimum continuous thickness did provide increased transmittance and decreased sheet resistances for the TAT, TAZ, and TAOT configurations. However, their transmittance spectra were still inferior to that of the TAOZ configuration due to increased reflection, especially in the spectral range above 600 nm, with increases in the thickness of Ag and Ag_(O)_ TEs (Fig. 5b). The transmittance of TAOZ was far superior to that of 160 nm a-ITO TE coated with the 20 nm TiO_2_ ETL (T-ITO). When the transmittance of the TAOZ configuration was further controlled by changing the thickness of the TiO_2_ ETL, the transmittance clearly red-shifted with the increasing ETL thickness (Fig. 5c). The TAOZ configuration utilizing the 20 nm TiO_2_ ETL exhibited the highest transmittance in the spectral range that is absorbed by PSCs. The negative influence of the thickness of the bottom ZnO layer on the transmittance of the TAOZ configuration provided a clear reason for choosing the thickness of 5 nm, which was just over the minimum possible thickness to form a continuous layer (Fig. S8†). Notably, a disparity was found between the thicknesses of the Ag_(O)_ TE required to maximize the optical transmittance of the Ag_(O)_ TE and to achieve a pinhole-free morphology of the TiO_2_ ETL. The change in the transmittance spectrum of the TAOZ configuration as a function of Ag_(O)_ thickness clearly demonstrated that the transmittance was maximized at *ca.* 6.0 nm and then abruptly decreased with the further increasing thickness (Fig. 5d). However, the Ag_(O)_ TE needed to be at least 7.5 nm thick to attain a pinhole-free TiO_2_ ETL. The results indicated that transmittance of the TAOZ configuration was far superior to the transmittances of Ag and Ag_(O)_ layers deposited on PET substrates without oxide layers (Fig. S9†). This superior transmittance was achieved by ensuring optical matching between the completely continuous, pinhole-free Ag_(O)_ layer and the bottom/top oxide layers.

**Fig. 6:**
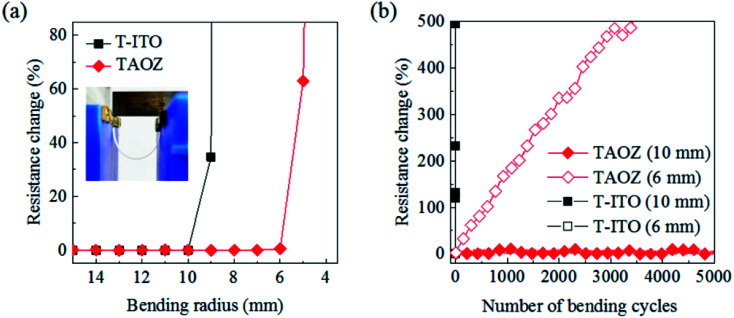
Mechanical flexibility of TAOZ and T-ITO fabricated on flexible PET substrates. (a) Change in the electrical resistance of 20T-7.5AO-5Z (TAOZ) and 20T-160ITO (T-ITO) as a function of the bending radius of PET substrates, which were subjected to bending with successively decreasing radii in irreversible bending tests. The inset shows a photograph of the bending test system. (b) Change in the electrical resistance of TAOZ and T-ITO as a function of the number of bending cycles in reversible bending tests. The PET substrates were cyclically bent using the two minimum bending radii of 6 and 10 mm determined in the irreversible bending tests (a).

After optimizing the thicknesses of the TE and ETL, the mechanical stabilities were examined for 20 nm TiO_2_/7.5 nm Ag_(O)_/5 nm ZnO and 20 nm TiO_2_/160 nm ITO (20T-160ITO) configurations. Each layer is hereafter labeled with its thickness in nanometers. The configurations were irreversibly deformed on the PET substrates with a bending radius decreasing from 15 mm to 4 mm (Fig. 6a). The 20T-7.5AO-5Z configuration exhibited a catastrophic increase in resistance at bending radii of less than 6 mm due to the abrupt development of microscopic cracks across TAOZ, whereas the 20T-160ITO configuration exhibited such electrical failure at gentler bending radii of less than 10 nm. Furthermore, TAOZ and T-ITO were subject to cyclic bending tests by reversibly deforming the PET substrates. Specifically, the PET substrates were bent repeatedly with two different minimum bending radii of 6 mm and 10 mm (Fig. 6b). The results confirmed that mechanical flexibility of TAOZ was superior to that of T-ITO. The resistance of TAOZ increased by less than 11%, even after 5000 bending cycles with a bending radius of 10 mm. Thus, the mechanical flexibility of TAOZ was far superior to that of T-ITO, which exhibited an immediate resistance increase at the same bending radius. The mechanical flexibility of TAOZ can be ascribed to both the ductility of the ultrathin metallic Ag_(O)_ TE and its pinhole-free morphology. Pinholes have often been suspected as the initiation sites for microscopic cracks under mechanical stress.^50^

**Fig. 7:**
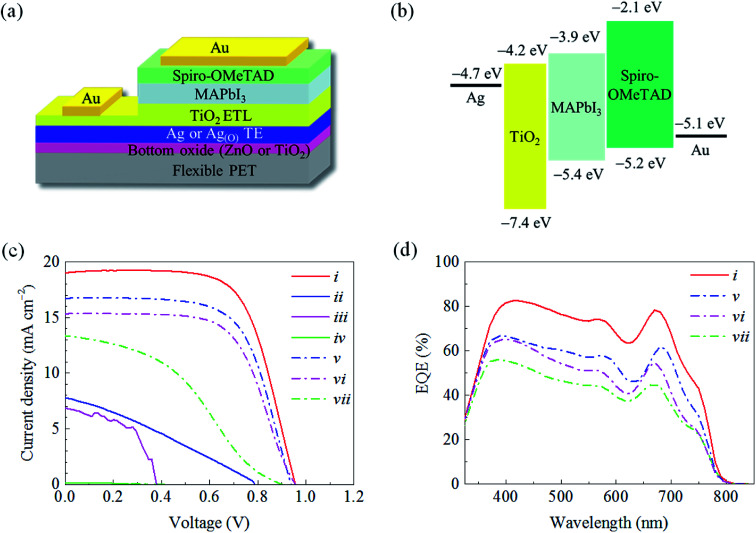
Photovoltaic performance of flexible PSCs fabricated on PET substrates with different OMO configurations. Schematic illustrations of (a) the structural architecture of PSCs used in the present study and (b) the corresponding energy level diagram. Comparison of (c) current density–voltage (*J*–*V*) characteristics and (d) EQE spectra of flexible PSCs utilizing different OMO configurations: (i) TAOZ with a 7.5 nm Ag_(O)_, (ii) TAOT with a 7.5 nm Ag_(O)_, (iii) TAZ with a 7.5 nm Ag, (iv) TAT with a 7.5 nm Ag, (v) TAOT with a 12 nm Ag_(O)_, (vi) TAZ with a 12 nm Ag, and (vii) TAT with a 12 nm Ag. The thickness of all TiO_2_ ETLs was set to 20 nm, while the thickness of all bottom oxide layers was set to 5 nm.

Flexible PSCs were fabricated with a device configuration of Au/2,2′,7,7′-tetrakis(*N*,*N*-di-*p*-methoxyphenylamine)-9,9′-spirobifluorene (spiro-OMeTAD)/methyl-ammonium lead iodide (MAPbI_3_)/OMO/PET with an emphasis on matching work function (Fig. 7a and b). Notably, the optimized 20T-7.5AO-5Z configuration provided a PCE of *ca.* 11.2%, which greatly surpassed those of the other OMO configurations for flexible PSCs fabricated on PET substrates (Table 1). This result effectively demonstrates the significant and positive influences of the absence of pinholes in the TiO_2_ and Ag_(O)_ layers on the photocurrent conversion performance of the PSCs utilizing the TAOZ configuration. The current density–voltage (*J*–*V*) characteristics (Fig. 7c) and external quantum efficiency (EQE) of PSCs (Fig. 7d) were seriously affected by the transmittance spectra of OMOs. Although hysteresis might be inevitable in planar-type PSCs due to the clear charge accumulation in the photoactive MAPbI_3_ layer,^51,52^ the high transmittance of the TAOZ configuration provided the highest EQE spectrum and current density. Meanwhile, the PSCs utilizing all OMOs besides TAOZ were hampered by an anomalous decrease in the PCE or even complete failure (*i.e.*, PCE = 0), which can be attributed to the strong current leakages that are believed to originate in the pinholes in the TiO_2_ ETLs. The strong detrimental influence of pinholes in TiO_2_ ETLs on the performance of the PSCs was alleviated by increasing the thickness of the Ag and Ag_(O)_ in the OMOs. The Ag and Ag_(O)_ TEs with a thickness of 12 nm exhibited a continuous layer morphology and thus significantly decreased the presence of pinholes in the TiO_2_ ETLs subsequently grown on the TEs. The performance of PSCs utilizing the 12 nm-thick Ag and Ag_(O)_ TEs dramatically improved, which could be attributed only to the decrease in pinholes, although their PCEs were still lower than that of the PSC utilizing the TAOZ configuration with the optimized 7.5 nm Ag_(O)_ TE because of their inferior optical transmittances. These results indicated that simultaneously achieving an ultralow optical loss and a pinhole-free morphology by improving the wetting of Ag_(O)_ directly enhanced the performance of flexible PSCs utilizing the TAOZ configuration. Furthermore, the photovoltaic performance that could be reached utilizing the optimized TAOZ configuration was still inferior to that of conventional PSCs utilizing crystalline TiO_2_ and TCO deposited on rigid glass at elevated temperatures. The limitations of TAOZ are mainly ascribed to the amorphous nature of TiO_2_ deposited at room temperature, exhibiting its inherent limitation for electron transport efficiency. However, it was deposited at this temperature to avoid thermally deforming its PET substrate. Nonetheless, this amorphousness negatively contributed to the photovoltaic performance of flexible PSCs by causing a decrease in the fill factor (FF) and an increase in the series resistance.

**Table 1:** Photovoltaic performance of flexible PSCs with different OMO configurations^a^

OMO	*J*_sc_ [mA cm^−2^]	*V*_oc_ [V]	FF [%]	*R*_s_ [Ω cm^2^]	*R*_sh_ [Ω cm^2^]	PCE [%]
(i) TAOZ	18.5 ± 0.5	0.94 ± 0.02	63.9 ± 7.5	15 ± 6	699 ± 503	11.2 ± 1.5
(ii) TAOT	5.3 ± 1.8	0.77 ± 0.05	27.9 ± 3.1	123 ± 47	285 ± 119	1.2 ± 0.5
(iii) TAZ	6.3 ± 0.6	0.35 ± 0.07	42.4 ± 10.9	26 ± 6	441 ± 425	1.0 ± 0.4
(iv) TAT	—	—	—	—	—	—
(v) TAOT	16.4 ± 0.3	0.92 ± 0.02	59.0 ± 4.7	23 ± 8	1867 ± 438	9.0 ± 1.0
(vi) TAZ	15.0 ± 0.3	0.95 ± 0.01	61.8 ± 2.6	30 ± 10	2507 ± 951	8.9 ± 0.4
(vii) TAT	13.7 ± 0.4	0.88 ± 0.02	40.6 ± 2.2	75 ± 19	423 ± 48	4.9 ± 0.4
T-ITO	17.7 ± 0.4	0.96 ± 0.01	51.6 ± 3.8	20 ± 4	636 ± 369	8.8 ± 0.6

a *J*_sc_, short-circuit current density; *V*_oc_, open-circuit voltage; FF, fill factor; *R*_s_, series resistance; *R*_sh_, shunt resistance; PCE, photocurrent conversion efficiency. Each parameter was averaged for 6 cells, fabricated using each OMO type. Data are presented as the average plus or minus the standard deviation.

## Conclusions

The main technical issues in fabricating highly efficient, flexible PSCs on heat-sensitive polymer substrates are related to the performance limitations of TE and ETL layers fabricated at room temperature, whereas in conventional PSCs based on rigid glass substrates, such problems are simply prevented by simple fabrication and/or annealing processes at higher temperatures to obtain a highly efficient, pinhole-free ETL on oxide-based TEs. Here, a thorough investigation has been carried out to develop an OMO configuration possessing both excellent optoelectrical and electron transport performance, in addition to strong mechanical flexibility. As a result, we report a technique to simultaneously achieve these multiple requirements by successfully fabricating (i) a completely continuous Ag_(O)_ TE at a highly decreased thickness of 7.5 nm, which ensures ultralow optical and electrical losses, and (ii) a pinhole-free TiO_2_ ETL on the Ag_(O)_ TE, which ensures efficient electron transport with no notable electron leakage. The continuous, pinhole-free Ag_(O)_ TE was deposited with dramatically improved wetting on the bottom ZnO layer. The absence of pinholes in the Ag_(O)_ TE directly suppressed the formation of pinholes in the subsequently deposited TiO_2_ ETL; such pinholes are generally believed to be the main source of current leakage. The excellent performance of the optimized TAOZ configuration and its TE and ETL was confirmed by the high PCE value of a flexible PSC fabricated on a heat-sensitive PET substrate at processing temperatures of no more than 100 °C. The highly detrimental effects of pinholes in both TE and ETL layers on the performance of PSCs were demonstrated by serious decreases in the efficiencies of PSCs with the increasing presence of pinholes in the TE and ETL layers in different OMO configurations.

## Experimental

### Fabrication

OMOs composed of Ag, Ag_(O)_, TiO_2_, and ZnO were fabricated at room temperature on 125 μm-thick PET (Panac Co., Ltd.) substrates with different structural configurations using a magnetron sputtering system (A-tech System Co.). These multilayer configurations were prepared by sequential sputtering processes without breaking the vacuum conditions. The sputtering processes were initiated once the chamber was evacuated to a base pressure of 7 × 10^−7^ torr. ZnO and TiO_2_ layers were deposited by sputtering 4-in stoichiometric ZnO and TiO_2_ targets (Applied Science Co.), respectively, at a radio-frequency power of 200 W and a working pressure of 3 × 10^−3^ torr, which was attained under an Ar atmosphere flowing at 60 sccm. The thicknesses of the bottom oxide layers were fixed at 5 nm, while the thicknesses of the top oxide layers were varied between 10 and 20 nm. The ITO sputtering conditions using a 4-in ITO target with 10 wt% Sn (Applied Science Co.) were identical to those of the oxide layers. Ag was deposited by sputtering a 4-in pure Ag target (Applied Science Co.) at a direct-current power of 50 W and a working pressure of 3 × 10^−3^ torr under an Ar atmosphere flowing at 45 sccm. Ag_(O)_ was deposited under conditions equivalent to those of Ag sputtering except for the mixed atmosphere of Ar and O_2_ at a flow rate of 45 : 4 sccm, which generated a reactive sputtering process. The sputtering system and process conditions are demonstrated in detail elsewhere.^35^

PSCs were fabricated on PET substrates using different OMO structural configurations: TiO_2_/Ag/TiO_2_, TiO_2_/Ag/ZnO, TiO_2_/Ag_(O)_/TiO_2_, and TiO_2_/Ag_(O)_/ZnO. Fabrication was initiated by exposing the OMOs coated on PET substrates to an ultraviolet (UV)-ozone environment for 20 min prior to coating the perovskite precursors on the OMOs. The MAPbI_3_ perovskite precursor solution was prepared by dissolving 0.461 g of PbI_2_ and 0.159 g of CH_3_NH_3_I (MAI) in a mixed solution of 71 μl of dimethyl sulfoxide (DMSO, anhydrous, 99.9%, Sigma-Aldrich) and 635 μl of *N*,*N*-dimethylformamide (DMF, anhydrous, 99.8%, Sigma-Aldrich). Then, MAPbI_3_ was deposited from the precursor solution by spin coating followed by a thermal treatment. Specifically, during the spin coating at 4500 rpm for 25 s, the precursor solution was mixed with 700 μl of diethyl ether, which was added dropwise. The spin-coated film was sequentially annealed at 60 °C for 1 min and 100 °C for 5 min. The HTL was prepared by dissolving 72.3 mg of spiro-OMeTAD in a mixture of 1 ml of chlorobenzene, 28.8 μl of 4-*tert*-butylpyridine (96%, Sigma-Aldrich), and 17.5 μl of Li-bis(trifluoromethanesulfonyl) imide (Li-TFSI), which was prepared by dissolving 520 mg of Li-TFSI in 1 ml of acetonitrile (99.8%, Sigma-Aldrich). The HTL was spin-coated on the MAPbI_3_ layer at 4000 rpm for 20 s. Finally, a top electrode based on Au with a thickness of 100 nm was deposited on the HTL by thermal evaporation with a device area of 0.075 cm^2^. A thin TiO_2_ layer between the Au rear contact and the Ag TE, as shown in the structural architecture of the PSCs (Fig. 7a), was applied purely to avoid any deterioration in the electrical conductivity due to oxidation of the TE. However, the thickness of the TiO_2_ layer was selected to be as low as 5 nm to minimize any detrimental increase in the series resistance, due to the insertion of the TiO_2_ layer.

### Characterization

The morphologies of the Ag and Ag_(O)_ TEs, which evolved from nanoclusters to agglomerates, and the TiO_2_ ETLs were investigated by capturing top-view and cross-sectional high-resolution images using ultrahigh-resolution FE-SEM (Hitachi High-Technologies Co., S-5500) at the Korea Basic Science Institute (KBSI, Jeonju, Republic of Korea). The distribution of nanoscopic pinholes in the TiO_2_ ETLs in different OMO structural configurations was determined using AFM (Bruker, Nanoscope V Multimode 8) in the conductive mode at a 600 mV bias. The surface area of pinholes was determined from the FE-SEM images using image processing software (Nikon, NIS-Elements Basic Research). The nominal thicknesses of the metal and oxide layers were determined either using X-ray reflectivity (Philips, X'pert Pro-MRD) at the KBSI in Daegu, Republic of Korea, or surface profiling (Bruker, Dektak XT) measurements at the Korea Institute of Materials Science (KIMS). The dose of oxygen was determined using XPS (Escalab 200 R, VG Scientific) at the Electronics and Telecommunications Research Institute (Daejeon, Republic of Korea). The crystallography of TiO_2_ and ZnO was characterized using XRD (Empyrean, PANalytical) at the KBSI Daegu. The total (specular + diffusive) transmittance and reflectance spectra were determined using UV-visible spectrophotometry (Agilent Technology, Cary 5000) in the visible spectral range of 330–800 nm. The absorbance spectra of the OMO structures were determined by ascertaining the transmittance and reflectance spectra. The sheet resistances of OMOs and ITOs were determined by averaging the measurements from three different samples with dimensions of 2.5 × 2.5 cm^2^ using a four-point probe (Hitachi Chemical Co., MCP-T600). The mechanical bending tests were carried out using a homemade bending test system by tracking the increase in the resistance of OMOs and ITOs as a function of the bending radius of PET substrates coated with OMOs and ITOs. The bending radius was decreased to 4 mm to subject the samples to increased tensile stresses. The structural reliability of the samples against repeated deformation was also determined by measuring the change in resistance as the samples were bent cyclically up to 5000 times. The *J*–*V* characteristic of PSCs was measured using a source measurement unit (Keithley 2400 SourceMeter) with a xenon lamp under one-sun conditions and a light intensity of 100 mW cm^−2^. The light source was calibrated using a modulated silicon solar cell that was certified by the Korea Institute of Energy Research (Taejon, Republic of Korea). The delay between each voltage step was fixed at 200 ms. The EQEs were determined using a Solar Cell Quantum Efficiency Measurement System (PV Measurement) over the wavelength range of 330–800 nm, and photo signals from the whole range were calibrated with photodiodes.

## Conflicts of interest

There are no conflicts of interest to declare.

## Supplementary Material

RA-009-C9RA00042A-s001
