# Supplementary material for: Pinhole-free TiO2/Ag(O)/ZnO configuration for flexible perovskite solar cells with ultralow optoelectrical loss
Source: RSC Adv. 2019 Mar 19;9(16):9160–70. doi: 10.1039/c9ra00042a (PMC9062062; doi:10.1039/c9ra00042a)
Supplement: RA-009-C9RA00042A-s001 [file RA-009-C9RA00042A-s001.pdf]

Electronic Supplementary Material (ESI) for RSC Advances.
This journal is © The Royal Society of Chemistry 2019

Electronic Supplementary Information

Pinhole-free TiO_2/$Ag_{(O)}$/ZnO Configuration for Flexible Perovskite Solar Cells with Ultralow Optoelectrical Loss

Eunwook Jeong,‡[a,b] Soohyun Bae,‡[c]* Jong Bae Park,[d] Seung Min Yu,[d] Donghwan Kim,[c] Hae-Seok Lee,[e] Jongjoo Rha,[a] Young-Rae Cho[b]* and Jungheum Yun[a]*

[a] Surface Technology Division, Korea Institute of Materials Science, Changwon, Gyeongnam 51508, Republic of Korea; Fax: +82-55-280-3570; Tel: +82-55-280-3515; E-mail: jungheum@kims.re.kr,

[b] Department of Materials Science and Engineering, Pusan National University, Busan 46241, Republic of Korea; Tel: +82-51-510-2389; E-mail: yescho@pusan.ac.kr

[c] Department of Materials Science and Engineering, Korea University, Seoul 02841, Republic of Korea; Tel: +82-2-3290-3713; E-mail: ramun16@korea.ac.kr

[d] Jeonju Center, Korea Basic Science Institute, Jeonju, Jeonbuk 54907, Republic of Korea

[e] KU-KIST Green School, Graduate School of Energy and Environment, Korea University, Seoul 02841, Republic of Korea

*Corresponding authors: E-mail: jungheum@kims.re.kr, ramun16@korea.ac.kr, yescho@pusan.ac.kr

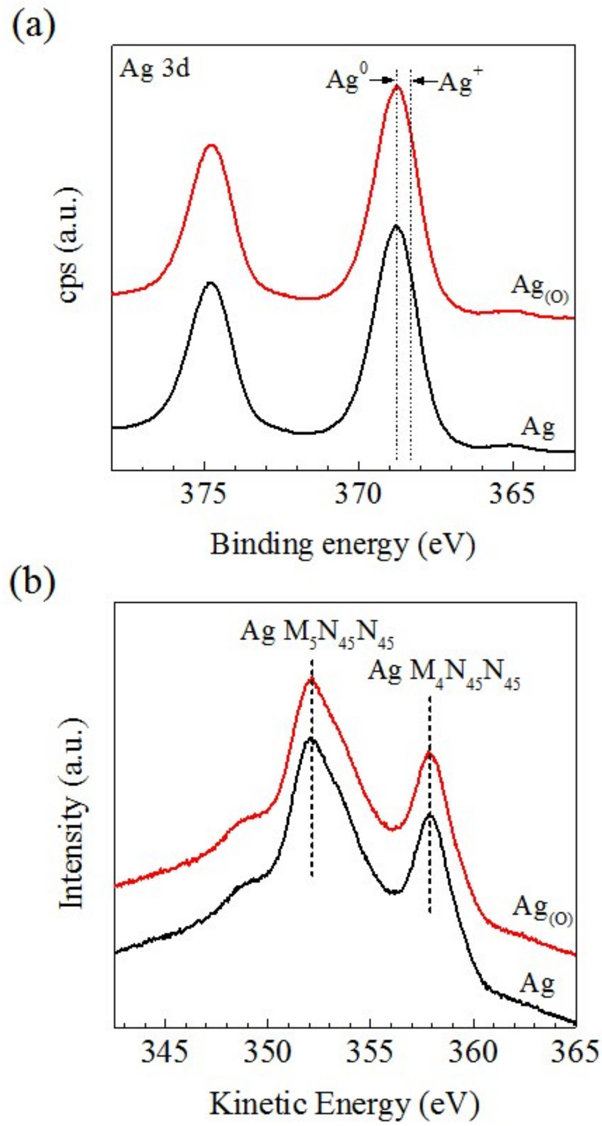

Fig. S1. Comparison of the chemical features of Ag and $Ag_{(O)}$. (a) X-ray photoelectron profiles of Ag 3d core level spectra and (b) Ag MNN Auger spectra measured from ca. 20-nm Ag and $Ag_{(O)}$ layers deposited on Si wafers.

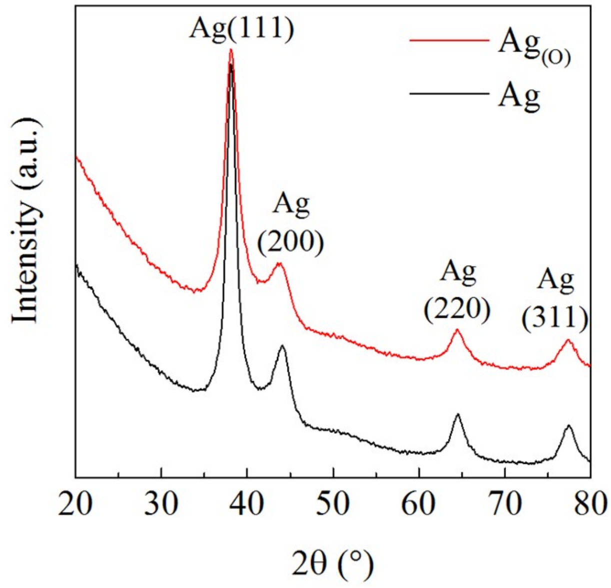

Fig. S2. Comparison of the crystallographic features of Ag and $Ag_{(O)}$. XRD patterns of 2θ scan measured for ca. 9-nm Ag and $Ag_{(O)}$ TEs sandwiched between ca. 15-nm SiO_2 layers.

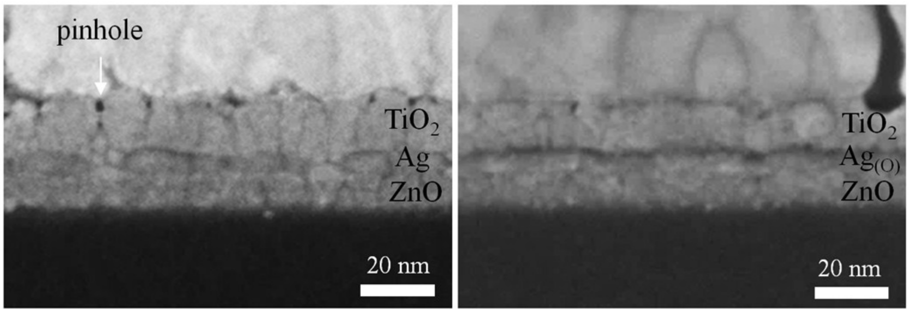

Fig. S3. Cross-sectional FE-SEM images of the pinhole distribution in TAZ (left) and TAOZ (right), which are composed of 20-nm top TiO_2 ETLs, either 7.5-nm Ag or $Ag_{(O)}$ TEs, respectively, and 15-nm bottom ZnO layer.

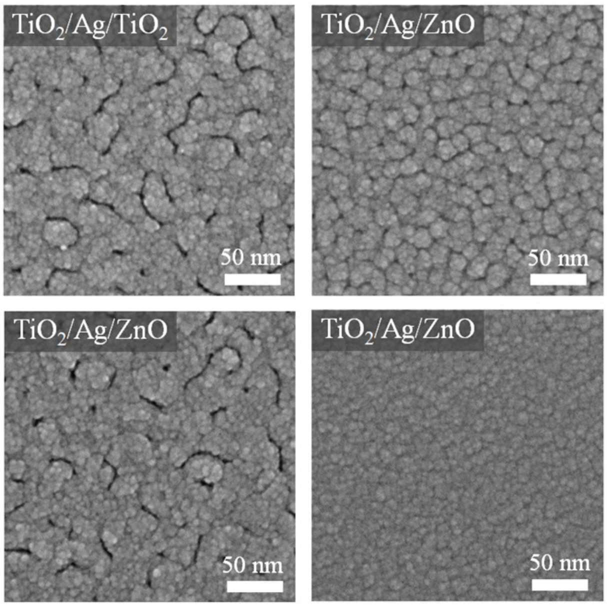

Fig. S4. Planar FE-SEM images of 10-nm top TiO_2 ETLs in different OMO configurations: TiO_2/Ag/TiO_2 (TAT), TiO_2/Ag/ZnO (TAZ), TiO_2/$Ag_{(O)}$/TiO_2 (TAOT), and TiO_2/$Ag_{(O)}$/ZnO (TAOZ) using 7.5-nm Ag and $Ag_{(O)}$ TEs and 5-nm bottom oxides.

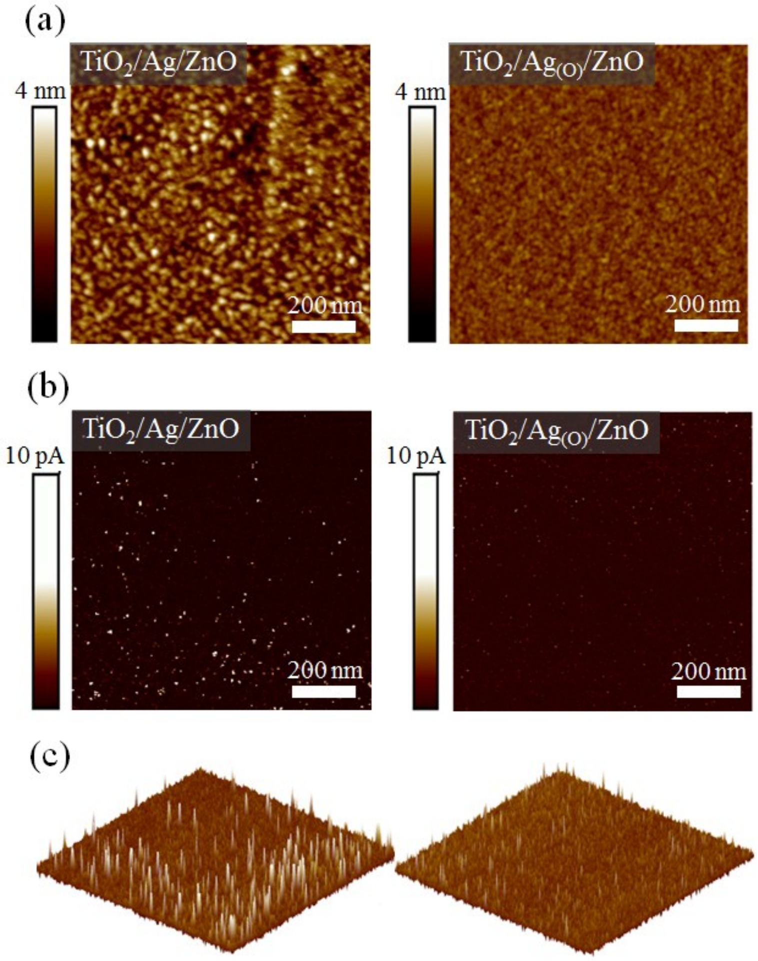

Fig. S5. Current leakage distribution through nanoscopic pinholes in 10-nm top TiO_2 ETLs. (a) 2D morphological scan images of 10-nm top TiO_2 ETLs of TiO_2/7.5-nm Ag/5-nm ZnO (TAZ) and TiO_2/7.5-nm $Ag_{(O)}$/5-nm ZnO (TAOZ) determined using tapping-mode AFM. (b) 2D and (c) 3D current scan images of the ETLs determined using conductive-mode AFM.

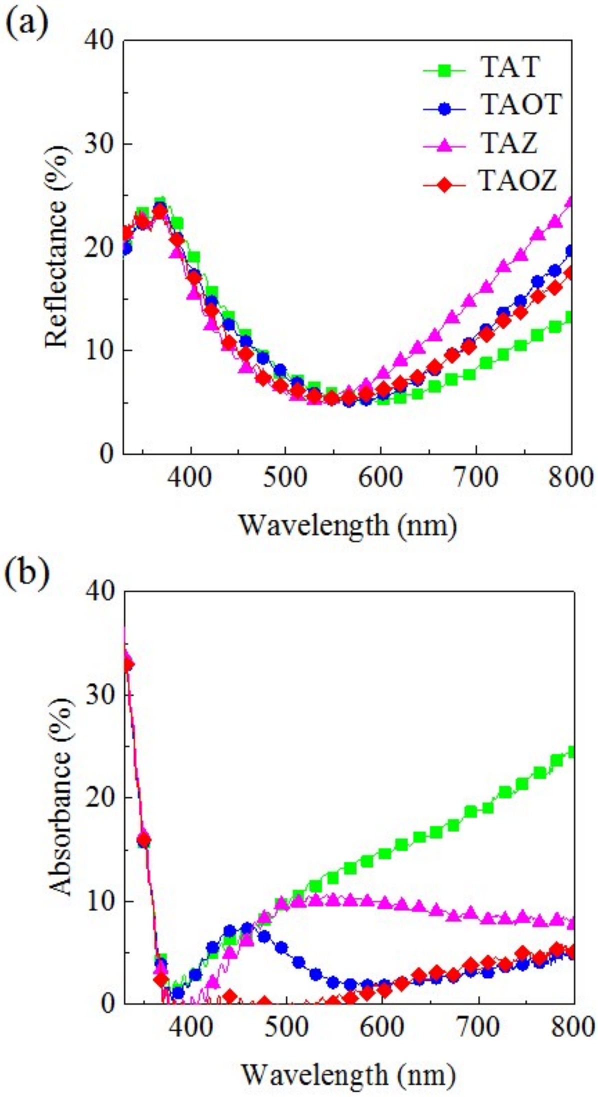

Fig. S6. Optical characteristics of OMOs. (a) Reflection and (b) absorbance spectra of TAT, TAOT, TAZ, and TAOZ using 7.5-nm Ag or $Ag_{(O)}$ TEs, corresponding to the conditions given in Fig. 5b. The absorbance spectra were determined by the formula absorbance = 100 − (transmittance + reflectance).

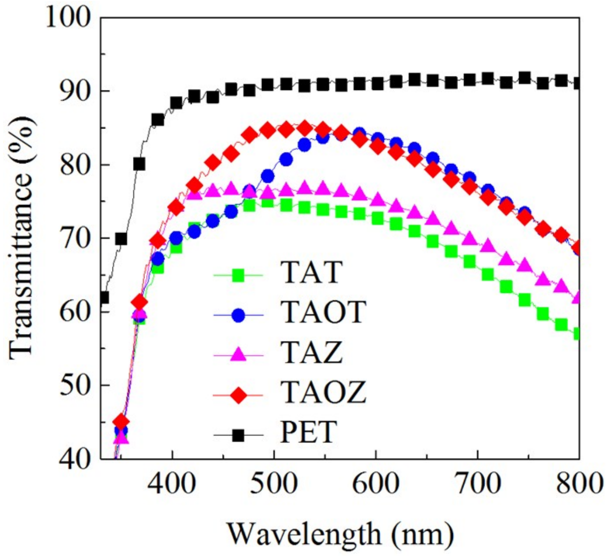

Fig. S7. Transmittance spectra of PET and OMOs using 7.5-nm Ag or $Ag_{(O)}$ TEs, corresponding to the conditions given in Fig. 5b. The transmittance spectra of OMOs include the transmittance loss due to their PET substrates.

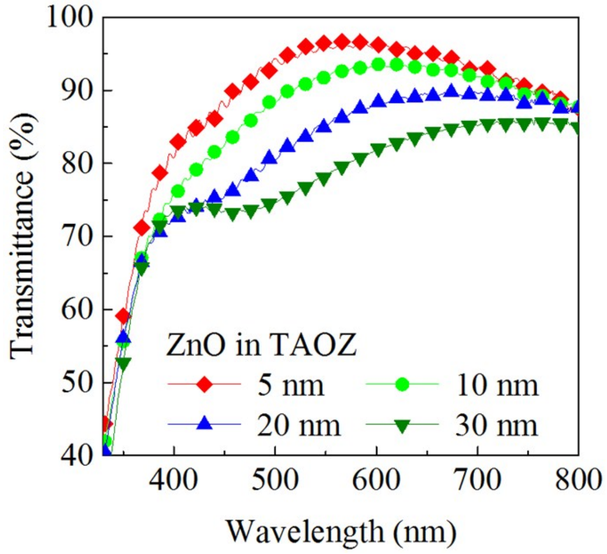

Fig. S8. Transmittance spectra of 20-nm TiO_2/7.5-nm $Ag_{(O)}$/ZnO (TAOZ) with bottom ZnO layers of various thicknesses. The transmittance of the PET substrates was subtracted from the transmittance spectra of the TAOZ configuration.

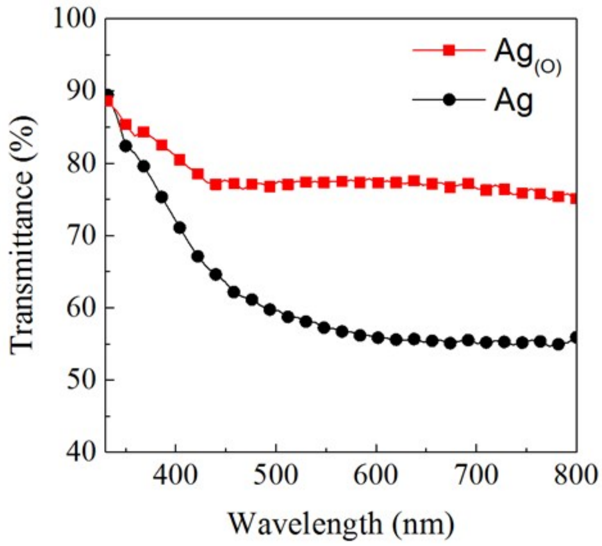

Fig. S9. Transmittance spectra of 7.5-nm Ag and $Ag_{(O)}$ layers deposited on PET substrates without oxides. The transmittance of the PET substrates was subtracted from the transmittance spectra of the Ag and $Ag_{(O)}$ single films.
